# Mothers’ experiences of breast milk expression during separation from their hospitalized infants: a systematic review of qualitative evidence

**DOI:** 10.1186/s12884-024-06323-3

**Published:** 2024-02-10

**Authors:** Xuemei Li, Yongqi Li, Lin Qian, Peng Han, Haoxue Feng, Hui Jiang

**Affiliations:** 1 Shanghai First Maternity and Infant Hospital, School of Medicine, Tongji University, Shanghai, 200092 China; 2 School of Nursing, Naval Medical University, Shanghai, 200433 China; 3 Nursing Department, Shanghai East Hospital, Tongji University, Shanghai, 200120 China; 4 Shanghai Tenth People’s Hospital, School of Medicine, Tongji University, Shanghai, 200072 China; 5 Nursing Department, Shanghai First Maternity and Infant Hospital, School of Medicine, Tongji University, Shanghai, 201204 China

**Keywords:** Maternal separation, NICU, Breastfeeding, Breast milk expression, Experience, Qualitative study, Meta-aggregation

## Abstract

**Background:**

Mother-infant separation, which is occurring with an increasing incidence, is a barrier to direct breastfeeding. Owing to the importance of breast milk to hospitalized infants, mothers are actively encouraged to express milk during their infants’ neonatal intensive care unit (NICU) stay. However, mothers are often faced with a number of challenges in this process. There is a need to understand such mothers’ real-life experiences of breast milk expression to develop supportive strategies to reduce the burden on mothers and increase breastfeeding rates.

**Methods:**

A comprehensive search of 12 databases was conducted for relevant studies published from database construction to December 2022. All qualitative and mixed-method studies published in English and Chinese that reported on mothers’ experiences of human milk expression during separation from their hospitalized infants were included. Two reviewers independently conducted screening, data extraction, and quality appraisal, with disagreements resolved by a third reviewer. The process of searching followed the Preferred Reporting Items for Systematic Reviews and Meta-Analyses (PRISMA) recommendations. The JBI Qualitative Assessment and Review Instrument was used to assess study quality and the credibility of study findings. Meta-aggregation was performed to integrate the results.

**Results:**

This systematic review aggregated mothers’ experiences of milk expression during separation from their hospitalized infants. Database search yielded 600 records, of which 19 full-text documents were screened. Finally, 13 studies of good quality were included with data from 332 mothers across seven countries. A total of 61 primary findings with illustrations were extracted from the 13 eligible studies, the findings were generalized into 16 categories, and further were concluded as four synthesized findings: purpose and motivation, physical and emotional experiences, barrier factors, and coping styles.

**Conclusion:**

Mothers were driven by extrinsic motivation in their decision to express breast milk. They experienced physical exhaustion and many negative emotional feelings while expressing. This process was affected by numerous barriers. Social support was essential to the initiation and maintenance of milk expression. Medical staff and families should pay more attention to the mental health of mothers with infants in the NICU. Future research should incorporate strategies to cope with emotional responses and offer practical strategies for managing milk expression.

**Systematic review registration:**

[www.crd.york.ac.uk], identifier [PROSPERO 2022 CRD42022383080].

**Supplementary Information:**

The online version contains supplementary material available at 10.1186/s12884-024-06323-3.

## Background

Breastfeeding has been identified as the optimum method for feeding infants, with considerable evidence supporting the health and psychological advantages to the mother-infant dyad [1]. Both the World Health Organization (WHO) and United Nations International Children’s Emergency Fund (UNICEF) are advocates of the practice of rooming-in to allow mothers and infants to remain together 24 h a day and of helping mothers initiate breastfeeding within a half-hour of birth followed by exclusive breastfeeding for 6 months [2]. The WHO target for exclusive breastfeeding in the first 6 months is at least 50% by 2025 [3]. However, by 2018, only 44% of infants globally were initiating breastfeeding within 1 h of birth, and 40% of infants under 6 months were exclusively breastfed [4].

Increasing mother-infant separation has been recognized as an obstacle to successful breastfeeding [5]. It is estimated that 10–15% of infants are admitted to a neonatal intensive care unit (NICU) due to low birth weight (LBW), preterm, congenital anomalies, perinatally acquired infections, and other diseases [6]. The separation disrupts lactation and leads to a low rate of breast milk provision for hospitalized infants. A survey including 6997 infants in the NICU in the USA indicated that 52% of very low birth weight (VLBW) infants were on formula milk only upon discharge and only 6% of infants were discharged on exclusive human milk [7]. In China, only 3.7–20.0% of neonates in hospital are fed with breast milk [8].

Breastfeeding is viewed as a lifesaving medical intervention for hospitalized infants [9], and the priority is to expose them to exclusive human milk as soon as possible. Breast milk has significant benefits in that it not only provides nutrition but also, more importantly, immunological protection. It has been shown to improve feeding tolerance [10], decrease the risk of necrotizing enterocolitis [11], and have long-term benefits to the infants’ health such as lower risks of hypertension and obesity [12]. The provision of breast milk also provides mothers with an opportunity for closeness, interaction, and the provision of comfort and affection so that the mother-infant relationship can be promoted [13, 14]. Baby Friendly Hospital Initiative for Neonatal Units (neo-BFHI), aimed to improve breastfeeding support for preterm and ill infants in the NICU, also emphasizes the importance of early skin-to-skin contact and early initiation of breastfeeding, and recommends lactation counseling including demonstration of milk expression by hand or pump should be provided by trained staff [15]. A delay in initiating breastfeeding beyond the first hour after birth has been proven to increase the risk of neonatal mortality [16]. Therefore, mothers should be encouraged to actively stimulate lactation and initiate milk production through early and regular milk expression. Early initiation of milk expression can maintain milk supply, laying the foundation for future breastfeeding [17]. Mothers who desire to breastfeed have to express breast milk until their infants are discharged from the NICU [17]. Additionally, in most NICUs in some countries such as China, mothers are not allowed to visit or participate in caregiving, including breastfeeding when their babies are admitted to the NICU; milk expression is hence an expedient [18].

Breast milk expression refers to extracting milk from the breast by hand or pump before it is fed to an infant, and is not the same as direct breastfeeding [19]. In this process, mothers may be faced with many challenges, such as inadequate milk supply, breast engorgement, and delayed initiation of their milk supply [20, 21]. They view that milk expression is stressful and many mothers experience anxiety [22]. In addition, manual and breast pump expression is not as effective as suckling at the breast in stimulating and maintaining milk supply, and mothers who express breast milk may struggle to initiate and maintain milk expression [23]. Some mothers report that pumping is expensive, time-consuming, and unpleasant in comparison with direct breastfeeding [24]. A previous study demonstrated that mothers’ experiences played a pivotal part in the establishment and duration of milk expression and breastfeeding [18]. Some relevant qualitative studies are available, but systematic integration has not been performed. Therefore, we conducted a qualitative meta-synthesis to provide further and comprehensive insight into the understanding of mothers’ experiences of milk expression during separation from their hospitalized infants.

## Aims

This systematic review aimed to interpret mothers’ experiences and perceptions while expressing during separation from their hospitalized infants, through critically appraising and synthesizing the qualitative evidence. In particular, the review may deepen the understanding of mothers’ experiences of milk expression among families, health professionals, and policymakers. What’s more, it can aid the future development and implementation of targeted in-hospital strategies for milk expression.

## Methods

### Design

A systematic review of qualitative research studies using meta-aggregation was conducted. This systematic review protocol was registered with PROSPERO, an international prospective register of systematic reviews (ID CRD42022383080). The Enhancing Transparency in Reporting the Synthesis of Qualitative Research (ENTREQ) checklist (Supplementary Table 1) was used to report the process and results of synthesis, and enhance transparency [25]. The process of searching and screening for studies was tracked using the PRISMA flowchart.

### Search strategy

**Fig. 1:**
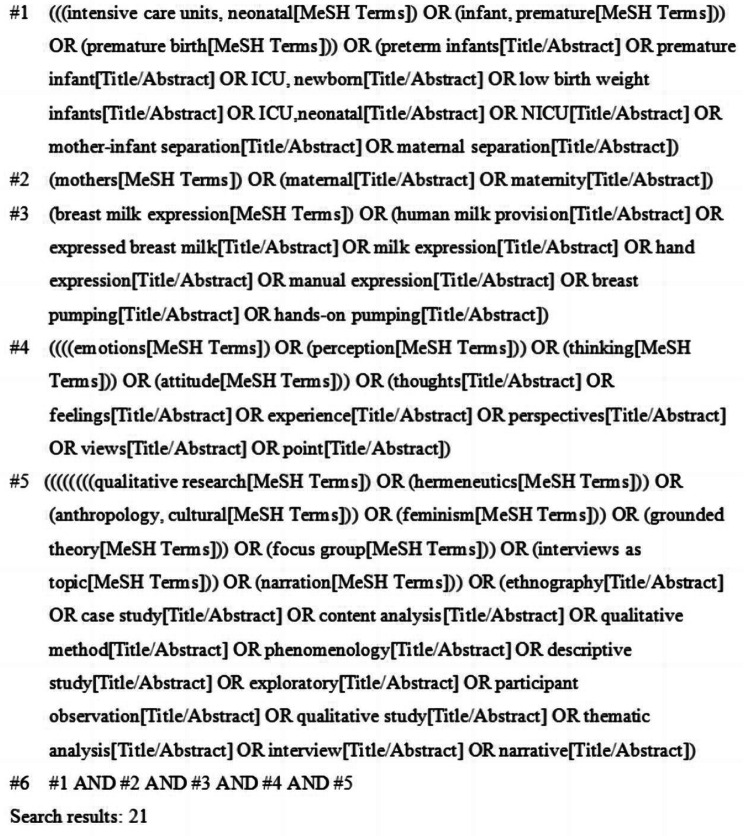
Search strategy in PubMed

We completed the entire search process for relevant published literature in three steps. First, an initial limited search via PubMed was conducted to analyze the index words and the derivatives of terms for studies related to mothers’ experiences with breast milk expression for their hospitalized infants, which helped identify search terms. Then, we systematically searched 12 electronic databases, including eight English language databases: PubMed, Web of Science Core Collection (via ISI Web of Science), MEDLINE (via ISI Web of Science), Cochrane Library, LWW (via OVID), CINAHL Complete (via EBSCO), Scopus, and ScienceDirect, and four Chinese databases: China National Knowledge Infrastructure (CNKI), Wanfang Database (CECDB), VIP Database, and China Biomedical Database (CBM). The decision to conduct our research only in these databases was based on an initial scoping search, which indicated that the most relevant studies for the topic could be found in these sources. The query included five groups of keywords and MeSH terms combined with Boolean operators: (1) (intensive care units, neonatal*), (infant, premature*), premature birth*, preterm infants, premature infant, (ICU, newborn), (ICU, neonatal), NICU, low birth weight infants, mother-infant separation, maternal separation; (2) mothers*, maternal, maternity; (3) breast milk expression*, human milk provision, expressed breast milk, milk expression, hand expression, manual expression, breast pumping, hands-on pumping; (4) emotions*, perception*, thinking*, attitude*, thoughts, feelings, experience, perspectives, views, point; (5) qualitative research*, hermeneutics*, (anthropology, cultural*), feminism*, grounded theory*, focus group*, interviews as topic*, narration*, ethnography, case study, content analysis, qualitative method, phenomenology, descriptive study, exploratory, participant observation, qualitative study, thematic analysis, interview, narrative. A separate search strategy was designed and optimized for different databases. Results were limited to journal articles written in English or Chinese and published before 1 December 2022. Finally, the references of each qualifying paper were searched manually to identify further relevant studies. The sample search strategy for PubMed is presented in Fig. 1.

### Inclusion and exclusion criteria

#### Inclusion criteria

Studies were included according to the following:

i) Participant (P). These were mothers who were separated from their babies that were admitted to a NICU for various reasons such as preterm and low birth weight.
ii) Interest of phenomena (I). Studies were included that focused on mothers’ experiences when they expressed breast milk by hand expression (manual expression), hand-powered pumping (hand pumping or manual pumping), electric-powered pumping (breast pumping), and hands-on pumping (combining hand expression and breast pumping).
iii) Context (Co). Included studies were those performed during mother-infant separation with the infants in the NICU.
iv) Study design (S). Qualitative research and mixed-method studies from which the qualitative part could be extracted were included. Studies were included that used any qualitative methodology, including but not limited to phenomenology, grounded theory, case studies, action research, ethnography, and feminist research.

#### Exclusion criteria

i) Studies with qualitative data that were analyzed using quantitative methods.
ii) Duplicate and unavailable full-text literature.
iii) non-English or Chinese literature.
iv) Research not published in peer-reviewed journals, case reports, conference proceedings, poster abstracts, and theses.
v) Systematic reviews and other reviews. We reviewed their references to identify possible relevant studies.

### Study selection

Study selection was tracked and reported using the PRISMA standards [26]. All records obtained in the initial search were imported into EndNote 20, and duplicate entries were removed. Then two trained reviewers (XL, YL) independently screened the literature according to the inclusion and exclusion criteria. Initially, titles and abstracts were assessed for eligibility. If eligibility was unclear after screening the abstract, the full text was read for further evaluation. The third reviewer (PH) was engaged in discussion if disagreements arose at any stage of the evaluation.

### Appraisal of methodological quality

A critical appraisal of the methodological quality was performed on the 13 included studies by two trained reviewers (XL, YL) independently, using the JBI Qualitative Assessment and Review Instrument [27]. When the evaluation results conflicted, the third researcher (PH) decided. Ten items were evaluated with “yes” (the study fulfilled the domain criteria), “no” (the study did not fulfill the domain criteria), “unclear” (the study’s adherence to certain domain criteria could not be conclusively proven), or “not applicable”. The reviewers finally included the articles that achieved a minimum of 60% “yes”, a criterion identified by the reviewers to guarantee the study showed acceptable quality. The quality of the study was considered acceptable if 60% of the items answered “yes”, good if 70–90% of the items answered “yes”, and high if 100% of the items answered “yes” [28].

### Data extraction and synthesis

The JBI meta-aggregation approach was used to extract and synthesize the data [27]. Meta-aggregation is grounded in the philosophical traditions of pragmatism and Husserlian transcendental phenomenology, which is the most transparent and widely accepted methodology of all available for qualitative study synthesis for constructing high-quality systematic reviews of qualitative research [27]. This approach accurately and reliably presents the findings and exemplar quotes from original study, categorizing similar phenomena together, and constructing synthesis statements [27].

First, dual data extraction was conducted, including the author, publication year, country or region, research aim, research design, method of data collection, sampling and data analysis, participants, and main research results of each qualified study. Second, study findings were extracted by fully grasping the meaning of each eligible study. A finding was defined as a verbatim extract of the author’s analytical interpretation of the data with illustrations. Two reviewers (XL, YL) independently evaluated the plausibility of each finding and identified them into three levels: (1) Unequivocal (U): the finding was supported by an accompanying illustration that was beyond reasonable doubt and therefore the finding was not open to challenge; (2) Equivocal (E): there was a lack of clear association between the finding and the accompanying illustration, and therefore the finding was open to challenge; (3) Not Supported (NS): the finding was not supported by the illustration. Only unequivocal and equivocal findings were included [27]. The extracted findings that possessed similarity in meaning or addressed a similar phenomenon together were summarized to form new categories. Ultimately, these categories were subjected to further synthesis to generate more comprehensive findings, known as synthesized findings. The process of data extraction and synthesis was conducted by two independent reviewers (XL, YL) based on discussions with the third author (PH).

## Results

### Search results

**Fig. 2:**
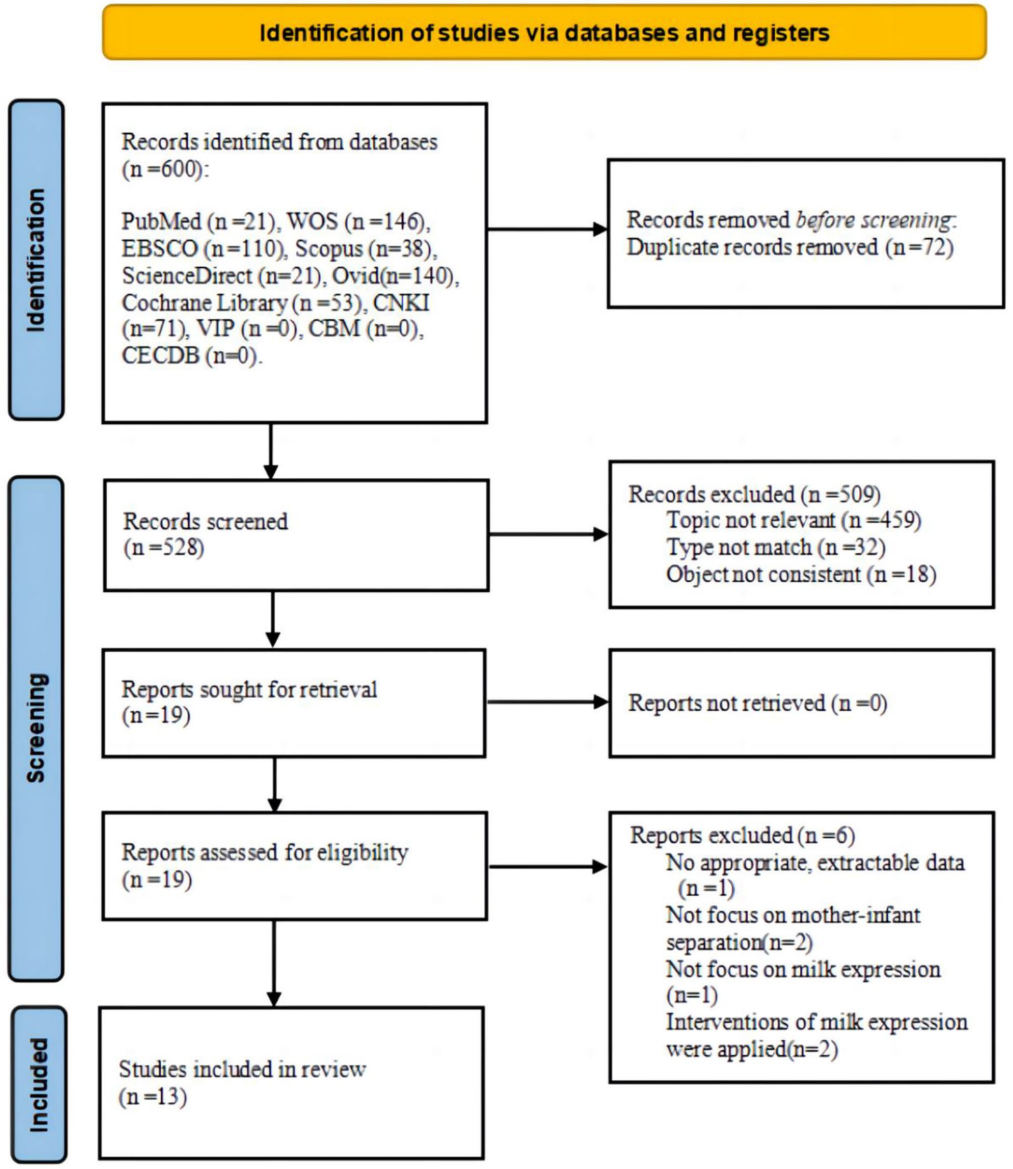
PRISMA flow diagram for article selection

The database search generated 600 records. Eliminating 72 duplicates left 528 unique records for screening. Then, by reading the titles and abstracts, 509 articles were excluded that were not relevant to the topic or subject, or were not qualitative research. Finally, 13 articles were included in the qualitative synthesis after reading the full text and quality appraisal. The detailed search and screening process is illustrated in Fig. 2.

### Methodological quality

All 13 eligible studies were of good quality. The results of the methodological quality appraisal are presented in Table 1.

**Table 1:** Methodological quality of the 13 included studies

Study^*^	Q 1^**^	2	3	4	5	6	7	8	9	10	Total percent of “yes” (%)
Brodsgaard et al. (2022) [29]	U	Y	Y	Y	Y	Y	Y	Y	Y	Y	90
Fernández Medina et al. (2019) [30]	Y	Y	Y	Y	Y	N	Y	Y	Y	Y	90
Yang et al. (2019) [18]	Y	Y	Y	Y	Y	N	Y	Y	Y	Y	90
Bujold et al. (2018) [31]	Y	Y	Y	Y	Y	N	N	Y	Y	Y	80
Parker et al. (2018) [32]	U	Y	Y	Y	Y	N	N	Y	Y	Y	70
Bower et al. (2017) [17]	Y	Y	Y	Y	Y	N	Y	Y	Y	Y	90
Froh et al. (2017) [33]	U	Y	Y	Y	Y	N	Y	Y	Y	Y	80
Rossman et al. (2017) [34]	U	Y	Y	Y	Y	N	Y	Y	Y	Y	80
Ikonen et al. (2016) [35]	U	Y	Y	Y	Y	N	Y	Y	Y	Y	80
Rossman et al. (2013) [36]	U	Y	Y	Y	Y	N	Y	Y	Y	Y	80
Hurst et al. (2013) [37]	Y	Y	Y	Y	Y	N	Y	Y	Y	Y	90
Sisk et al. (2010) [38]	U	Y	Y	Y	Y	N	Y	Y	Y	Y	80
Sweet. (2008) [39]	Y	Y	Y	Y	Y	N	Y	Y	Y	Y	90

*Critical appraisal (*n* = 10): Y = yes; N = no; U = unclear; NA = not applicable

**Question = Q, Critical appraisal questions for qualitative studies:

Q1: Is there congruity between the stated philosophical perspective and the research methodology?

Q2: Is there congruity between the research methodology and the research question or objectives?

Q3: Is there congruity between the research methodology and the methods used to collect data?

Q4: Is there congruity between the research methodology and the representation and analysis of data?

Q5: Is there congruity between the research methodology and the interpretation of results?

Q6: Is there a statement locating the researcher culturally or theoretically?

Q7: Is the influence of the researcher on the research, and vice-versa, addressed?

Q8: Are participants and their opinions adequately represented?

Q9: Are the research ethics according to the current criteria or for recent studies, and is there evidence of ethics approval by an appropriate body?

Q10: Are the conclusions drawn in the research report obtained from the analysis or interpretation of the data?

### Study characteristics

Study characteristics are presented in Table 2. All the studies were published after 2008 and were original articles. A total of 332 mothers were interviewed in the studies. Included studies were undertaken in China (*n* = 1), Denmark (*n* = 1), Spain (*n* = 1), Canada (*n* = 1), Finland (*n* = 1), Australia (*n* = 1), and the USA (*n* = 7). Study designs included qualitative exploratory approach (*n* = 1), interpretative approach (*n* = 1), phenomenological approach (*n* = 3), qualitative descriptive approach (*n* = 4), grounded theory (*n* = 1), mix-method study (*n* = 1), ethnography (*n* = 1), and a study described as qualitative without a specific approach (*n* = 1).

**Table 2:** Characteristics of the 13 included qualitative studies

Study	Country	Aim	Research method	No. of participants	Characteristics of participants	Results
Brodsgaard et al. (2022) [29]	Denmark	To explore the circumstances that affect lactation for mothers of premature infants and their significance	Qualitative exploratory approach Purposive sampling Semi-structured, individual interview Content analysis	16	Mothers of premature infants admitted to a university hospital NICU in the Capital region of Denmark	Three themes: 1. The birth preparation period has been interrupted. 2. Expressing human milk is essential for breastfeeding. 3. The motherhood journey encompasses breastfeeding.
Fernández Medina et al. (2019) [30]	Spain	To describe and understand the experiences of mothers of extremely preterm infants regarding barriers to providing their own milk during infant hospital stay in the NICU	Qualitative, interpretative design using Gadamer’s hermeneutic approach Convenience sampling Semi-structured interviews A modified form of the stages developed by Fleming et al.	15	Mothers of extremely preterm infants who were admitted to a level III NICU in the southeast of Spain	Two themes: 1. Unexpected and unusual lactation. 2. Providing mother’s own milk to a tiny infant in an unknown technological environment.
Yang et al. (2019) [18]	China	To understand Chinese mothers’ experiences of expressing breast milk for their preterm hospitalized infants and to identify the support they needed to establish and maintain an adequate milk supply	Husserl’s descriptive phenomenological approach Purposive sampling Semi-structured, individual interviews Thematic analysis	11	Mothers separated from their preterm infants admitted to three level III Beijing NICUs in 2017	Four themes: 1. Providing breast milk is a way to identify as a mother. 2. Perceptions and intentions. 3. Milk expression makes mothers exhausted. 4. Health professional support for breastfeeding is urgently needed.
Bujold et al. (2018) [31]	Canada	To document maternal experiences expressing human milk for their infant in the NICU as a closeness or separation experience, and to discover what factors gave rise to these perceptions	Qualitative descriptive approach Convenience sampling Audio recorded their thoughts and feelings with a smartphone application Thematic analysis	15	Mothers who were expressing while their infant was hospitalized in a level 3 urban Canadian NICU	Three themes: 1. Finding ways to cope with milk expression helps mothers feel close. 2. Being overwhelmed by the challenges and feeling separated. 3. Location and environment.
Parker et al. (2018) [32]	USA	To examine the perceived barriers and facilitators of providing milk for very preterm infants during the hospitalization among Hispanic and non-Hispanic black mothers	Grounded theory approach Purposive sampling Semi-structured interviews Systematic, iterative approach to data analysis	23	Mothers that initiated milk production for their very preterm infants in the NICU—12 English and Spanish-speaking Hispanic mothers and 11 non-Hispanic black mothers	Four themes pertaining to general experiences: 1. Breastfeeding intent impacts mothers’ success in providing milk throughout the hospitalization. 2. Pumping milk for a hospitalized infant is repetitive and exhausting, and does not elicit the same emotional connection as breastfeeding. 3. Hospital providers are an important source of support, when sufficient time is spent to address ongoing issues. 4. Providing milk creates a unique sense of purpose when mothers otherwise feel a lack of control. Four themes pertaining to the experiences of Hispanic and non-Hispanic black mothers: 1. Breastfeeding as a cultural norm influences mothers’ intent to initiate and continue breastfeeding. 2. Hospital staff are viewed as more supportive when interactions and treatment are perceived as racially/ethnically unbiased. 3. Hospital staff are viewed as more supportive when communication occurs in the primary language. 4. Mother-infant separation creates logistical challenges that negatively impact ongoing milk production.
Bower et al. (2017) [17]	USA	To describe mothers’ experiences expressing milk for their VLBW infants in a level 3 NICU	Phenomenological approach Purposive sampling Individual interviews and telephone interviews Colizzi’s seven-step phenomenological analysis	17	Mothers of VLBW at a local level 3 NICU in the southeastern United States	Three themes: 1. I was heartbroken. 2. Pumping is a full-time thing. 3. I literally sacrificed nights.
Froh et al. (2017) [33]	USA	To describe the breastfeeding experience of mothers of infants with congenital diaphragmatic hernia (CDH) cared for in the NICU	Qualitative descriptive approach Purposive sampling Phased interviews Content analysis	11	Mothers with an CDH infant were recruited from a large level 3 NICU in an urban, northeastern pediatric hospital	Six themes: 1. Hopeful for breastfeeding. 2. Latching on … to the pump. 3. We’ve already worked so hard. 4. Getting the hang of it—it’s getting easier. 5. A good safety net. 6. Finding a way that works for us.
Rossman et al. (2017) [34]	USA	To describe the human milk provision experiences, goals, and outcomes for teen mothers of LBW infants hospitalized in the NICU	A multi method study using a qualitative research design, survey, and infant medical records Purposive sampling In-depth, semi-structured individual interviews Content analysis	15	Teen mothers (12 black, 3 Hispanic) of LBW infants hospitalized in a tertiary NICU	Two themes: 1. Wanting to do the right thing. 2. Everything’s fine.
Ikonen et al. (2016) [35]	Finland	To describe mothers’ experiences with expressing breast milk for preterm or small for gestational age (SGA) infants	Qualitative descriptive approach Convenience sampling Internet-based questionnaire with open-ended questions Inductive content analysis	130	Mothers who required to express milk for their preterm or SGA infants admitted to a NICU	Four themes: 1. Managing the situation. 2. Looking forward to easier breastfeeding. 3. Managing daily life. 4. Managing feelings.
Rossman et al. (2013) [36]	USA	To describe the meaning of milk for mothers who are providing milk for their VLBW infants hospitalized in the neonatal intensive care unit	Qualitative descriptive approach Convenience sampling Semi-structured interviews Content analysis	23	Mothers of VLBW infants hospitalized in the level 3 NICU at a large Midwestern urban medical center	Two themes: 1. The healing properties of milk related to the infant. 2. The healing properties of milk related to the mother.
Hurst et al. (2013) [37]	USA	To understand the experience of mothers of hospitalized very preterm infants related to their daily pumping milk routine during the NICU stay	Medical ethnographic approach Purposive sampling Semi-structured interviews Thematic analysis	14	Mothers expressing their milk using hospital-grade mechanical pumps exclusively to provide their breast milk for their infants in the level 2 NICU in southeastern Texas	Three themes: 1. Becoming a “mother–interrupted.” 2. A paradoxical experience. 3. Negotiating.
Sisk et al. (2010) [38]	USA	To identify patterns of factors that supported or hindered initiation of breast milk expression and maintenance of breast milk production after the birth of a VLBW infant in a sample of US women with varied prenatal infant feeding intentions	Qualitative approach Purposive sampling Semi-structured, individual interviews Content analysis	32	Mothers who initiated breast milk expression after delivery of their LBW infants at the Sara Lee Center for Women’s Health NICU, Forsyth Medical Center.	Two themes: 1. Barriers and supports for initiation of breast milk expression. 2. Barriers and supports for maintenance of breast milk production.
Sweet. (2008) [39]	Australia	To understand the breastfeeding experience of parents of VLBW preterm infants	Interpretive phenomenological approach Purposive sampling Semi-structured, individual interviews Thematic analysis	10	Mothers who intended to breastfeed their preterm VLBW infants in the NICU	Five themes: 1. Breastfeeding is initially not a priority. 2. Being a “good” parent. 3. Breastfeeding as a marker of “good” motherhood. 4. “So much is taken out of your hands.” 5. “You have to do it”—expressing is not a choice.

### Results of meta-synthesis

A total of 61 primary findings with illustrations, classified as equivocal or unequivocal, were extracted from the 13 included studies. The findings were generalized into 16 categories based on their meaning. From the 16 categories, four synthesized findings were constructed: purpose and motivation, physical and emotional experiences, barrier factors, and coping styles. The main findings with illustrations and levels of credibility are presented in Supplementary Tables 2, and the detailed process of synthesis is reported in Supplementary Table 3.

#### Synthesized finding 1: purpose and motivation

##### To alleviate the feeling of guilt

Preterm birth was always an unexpected pregnancy outcome, interrupting the normal preparation for birth. Mothers were filled with a sense of guilt about the traumatic birth story, and they blamed themselves for delivering early. “*I couldn’t hold her in my uterus long enough*” [29]. Therefore, they readily accepted pumping milk to compensate for the growth process that was halted by premature birth: “*I’m doing for her outside what I couldn’t do for her in my womb*” [29]. By this means, the feeling of guilt was alleviated and they healed themselves.

##### For sake of infants’ health

Breast milk had a number of advantages, such as healing power like medicine and mitigating complications, which was extremely valuable for those children who were born prematurely and with LBW [29–32]. Breast milk was even so important that babies depended on it for survival: “… *in the beginning, a lifeline [breast milk] for the baby*” [32]. As a result, for the sake of their babies’ health, mothers resolutely decided to provide their milk regardless of their own needs: “*I’m also realistic and it’s not about me—it’s about what’s best for him so I’m willing to do whatever. It would be my first choice, but if it doesn’t work, I’ll still pump and give it to him in a bottle*” [33]. In turn, they were motivated to express more milk when they witnessed the positive impact of milk on their babies: “… *it’s [providing milk] doing something for him, it’s very rewarding and it’s sustaining his life. And he’s gaining weight, so it specially makes you feel good*” [29].

##### To create a bond with infants

Willingness to provide milk was also driven by perceptions that breast milk served as a bridge for mothers to sustain a continued biological connection with their hospitalized infants: “*I think the breast milk—it’s me. I feel connected ’cause my breast milk is a part of me. I mean, I’m makin’ this milk*” [29]. Also, the provision of milk symbolized an emotional connection: “*I was happy because I’ve always liked to breastfeed my kids, to me that’s a very beautiful experience*” [31]. Through the ritual of providing milk, they seemed to participate in care even if their infants were not with them: “*Even if I can’t be there for him every single day, I’m pumping still. So you feel like you’re doing something for him. I just don’t want anything else to get in the way of pumping*” [29].

##### As a symbol of maternal role

Apart from the preterm infants admitted to NICU, the only thing mothers could do was to provide expressed milk: “*Preterm infants are more fragile than full-term infants. The only thing mothers can do is to express more milk for them*” [18]. They viewed it as a symbol of a mother or even a useful mother. Two exemplary quotes follow: “*It’s the breasts starting to go up and milk oozing out that let me think I am a mother*” [18]; “*… pumping and giving him milk is like a way to feel like you are important for your child. You feel you’re a mother*” [34]. In addition, mothers held the view that a good mother should provide an adequate milk supply and a longer duration of breastfeeding, otherwise, they would become disappointed: “*Yeah, inadequate a bit. Like you weren’t doing what you were supposed to be doing, or getting, that you were a bit of a failure*” [35]; “*[to be] A better mum than anybody else. Certainly not a bad mum. I tried and I did what I could [lasting longer at breastfeeding than other mothers did] and, you know*” [35].

##### For the transition to breastfeeding

When separated from hospitalized infants, mothers could not feed their babies directly at the breast, instead expressing their milk if they held a personal breastfeeding goal [33–36]. Mothers perceived human milk expression as an essential foundation and a stepping stone to the goal of lactation: “*Pumping my milk, well it is part of the breastfeeding process*” [34]. In addition, they were optimistic about the future, looking forward to the eventual transition to breastfeeding, as one mother described: “*And maybe that will happen, and if it does that’s great. I mean, I’d love to see … hopefully I’ll have the opportunity to try when he’s ready to*” [33]. The optimistic feeling as a motivator inspired them to continue expressing milk: “*But hopeful … to hopefully get to that point, continuing to pump to keep my supply up for when she is ready to breastfeed. That’s a huge motivator in itself*” [33]. They believed that, in due course, they would ultimately realize breastfeeding: “*Beginning by expression was worth it. I was able to breastfeed when the time came for me to do it*” [32].

#### Synthesized finding 2: physical and emotional experiences

##### Physical exhaustion

Expressing milk every 2 to 3 h was the required frequency to establish and maintain the milk supply; consequently, most mothers suffered exhaustion, especially at night: “*… too tired at night. No matter how late at night, as long as the alarm clock sounds, I have to sit up to express milk … [it] needs perseverance*” [18]. Sometimes, they were so tired that they overslept and missed a milk expression session: “*Sometimes I skip ‘cause I just oversleep and be so tired*” [17]. Being quite exhausted, combined with the complexity of the pumping process [31] and without reminders from their infants [17], mothers had less motivation in the evenings and at night to express milk. As a participant said, “*That’s really when I feel the most alone, the furthest away. I don’t think there’s really anything to help at that hour. You need willpower to get up, express your milk. Then you tell yourself, I’m tired, why am I doing this?*” [34].

##### Feeling of bereavement

Owing to the preterm birth, the infants were usually transferred to the NICU during the immediate postpartum period. Not seeing their babies, mothers suffered the feeling of bereavement. They thought that their infants had not survived and felt unable to begin milk expression: “*In less than 10 minutes my infant was no longer inside me, and I had not heard anything … and there I was, alone … Why would I express milk if I had not even seen my baby was alive? I had pre-eclampsia and I did not feel well. I could not visit my infant until 30 hours later …*” [30].

##### Overwhelmed by stress

Mothers encountered a range of pressures from various origins while expressing breast milk. Essentially, the separation from the infants was the primary source of stress: “*So it wasn’t easy for me. I did get a little depressed. Sometimes I would leave the place [NICU] crying. Many people, or the nurses would say everything is fine, but as a mom, you don’t want to leave your children there. It’s hard to see them there … it was like dividing myself*” [31]. Given the benefits of milk for infants during their hospital stay, the continuous demands for mothers’ milk by healthcare professionals made mothers feel pressured and constrained: “*I felt a bit overwhelmed because I felt bad and also I did not have enough milk … and I had to force myself because the nurses said I had to have more milk … There was great pressure to know that my milk was of vital importance to my infant …*” [30]. Sometimes, mothers found it stressful to pump based on the schedule: “*Pumping is the pits. I’ll be honest—it is not fun. Three hours … it seems like a long time but it’s not, so there’s always the stress of, oh my god, it’s almost time … I’ve gotta pump*” [17]. The pressure had a side effect on the expressed milk volume [31]; in turn, the decrease in breast milk production further exacerbated feelings of stress among mothers: “*I’m not feeling very close, just feeling stressed out that I’m not producing enough milk*” [34].

##### Closeness and separation

Almost all mothers experienced varying degrees of closeness and separation feelings while expressing [34]. Several reported that they felt closeness when pumping next to the babies: “*I pump my milk next to my baby’s isolette. I’m able to look at my baby. It’s a nice moment for me, I feel very close to him*” [34]. Merely thinking about being with the infant, either before or after a visit, made some mothers feel close: “*I still feel close to my baby because I went to see him this morning … When I think of him … pleasant thoughts come to me. I know he’s okay … that he’ll have good milk*” [34]. In contrast, mothers did not think milk expression was the same as breastfeeding [31, 34]. They felt more separated while expressing: “*I’m at home pumping … I feel isolated from our infant because we’re not there. He’s over at the hospital and we’re here and it feels strange to be doing something for him when he’s not in our home*” [34].

##### A paradoxical experience

A paradox, defined as a situation or action having seemingly contradictory qualities, can be resolved in some manner, and is distinguished from contradiction [37]. The detachment from the infants represented by the pump, along with the benefits of breast milk to their infant’s health, led to a paradoxical reaction among mothers [37]. Mothers expressed a profound dislike of the pumping process but continued to provide milk to help their infants [29]. They tried to persuade themselves that their love for babies melted into the process of pumping milk: “… *how you’re trying to love your child even though you are attached to a machine … it is pretty difficult to describe*” [37]; “*I do look at the pump but I know it’s not my baby, but I think this is for my baby*” [37].

#### Synthesized finding 3: barrier factors

##### Physical condition

Sometimes, the physical condition of the mother-infant dyad increased the difficulties in initiating and maintaining milk expression. On the one hand, when complications occurred in preterm infants, mothers were reluctant to touch their infants, contributing to the existing problems with milk expression: “*The problem came when my infant began to have serious health problems … I missed the timetable to express milk … I did not want to touch him or have skin-to-skin contact, and this caused the milk supply to go down …*” [30]. On the other hand, the physical challenges mothers faced impeded the initiation of milk expression. If a mother were diagnosed with seizures, she would receive magnesium sulfate for treatment. Mothers stated that this medication caused them to have difficulty comprehending the pumping instructions or not to feel well enough to pump: “*Have you ever been on mag[nesium]? Oh my god, you’re loopy … I just didn’t care … I was just drunk*” [38]. Additionally, some mothers reported that they did not follow the required pumping frequency because of the fatigue induced by cesarean Sect. [38].

##### Perceptions and attitudes

Negative attitudes and incorrect perceptions held by mothers also hindered milk expression. Most mothers indicated either mild or no intention to express milk [18, 32, 38]—an exemplary quote follows: “*… didn’t want to breastfeed*” [18]. These placed blame on mothers for not having the correct knowledge, including, but not limited to, misunderstanding the nutrition of breast milk and formula [18], the quality of milk being influenced by daily diet [18], and decreasing milk supply over time [32]. Some mothers also worried breastfeeding would have a negative impact on their work, life, and body image [18]. Some mothers considered breastfeeding was initially not a priority—they were more concerned about their infants’ health than establishing breastfeeding [18, 35]: “*My main concern was him, you know, even though they say express or whatever, I was never in my room, I was always downstairs … and all I’m concerned about is him medically, how he is, you know, not my milk supply, you know*” [35]. Additionally, the negative comments and attitudes from maternal mothers stood in the way of expression [18, 39]: “*My parents asked me not to breastfeed my baby because formula feeding is easier than breastfeeding*” [18]. What’s more, nurses on the postnatal ward paid less attention to mothers separated from the infants than others with babies, and didn’t show them how to express breast milk: “… *because I didn’t have Joel with me, you don’t have the nurses and all the people kind of giving you all this information, they’re dealing, you know, with the breastfeeding issue [s] with the women whose babies are right next to them. Me without having my baby, I didn’t have, like, the nurses coming in and having chats to me, …, they just showed me the machine, what I had to do and that was the end of it*” [35].

##### Practical obstacles

During the process of expressing, mothers encountered many practical obstacles. Owing to the separation, lack of breast stimulation was the main barrier to milk supply [30]. Mothers complained that expression as scheduled was restrictive in daily life and they had to balance between life, work, and pumping [32, 38]: “*Daily life revolved around pumping, and I had to schedule all my plans according to it. It was also taxing on my sleep routine*” [32]. For the pumping equipment, mothers lamented that the demands of extreme hygiene increased the workload [32], and expression became more difficult without a suitable pumping machine and comfortable pumping environment [32, 38]. For some mothers who transported pumped milk to the hospital, the distance interrupted the pumping frequency: “*It was hard to stay on a pumping schedule traveling back and forth to the NICU*”. At the same time, they also faced logistical and financial challenges: “*I spent so much of my money on cabs, coming back and forth to the hospital, and I had to space out times. I didn’t have the money to go back and forth. I think that’s the worst part of them being in the hospital, is transportation. That’s it*” [31].

#### Synthesized finding 4: coping styles

##### Seeking social support

Most mothers considered information and tips from hospital professional providers as an important social support; however, they perceived hospital staff were busy and had limited time to offer adequate support and supervision [18, 30, 31, 34]: “*Professional books published by doctors and nurses would be very helpful*” [18]; “*… doctors and nurses are too busy … [and] one-on-one guidance is not feasible*” [18]. With lack of access to professional support, some turned to non-professional sources for support and information, but did not get suitable answers. As one mother explained, “*There are many people in the WeChat group, but no one answered my questions, unlike one-on-one guidance where you will definitely get an answer*” [18]. Additionally, encouragement and support from husbands also increased their motivation to persist in expression: “*He [husband] is encouraging me a lot. He supports and understands and appreciates what I’m doing for our child. This is for me the power to continue pumping*” [34]. Therefore, social support for milk expression was essential for mothers and was urgently needed.

##### Self-regulation and adaptation

Mothers were resilient in continuing expressing breast milk and coped with the challenges of milk expression through self-regulation. Some mothers reminded themselves of their goal of lactation using self-talk as a motivator: “*It’s motivating to remind myself that I’m doing this for her, when I see her and I know she’s going to drink this milk*” [34]. Most mothers purposefully thought of their infant while expressing: “*I like to think about my baby, it brings positive thoughts. I know … he will get good milk*” [34], whereas some utilized distractions to pass the time when expressing milk: “*I’m alone in a small room so I check my phone, check social media, or play games … finding ways to pass time because I need to pump for 15 minutes. But I will go back to my baby right after*” [34]. Mothers also expressed milk in the presence of the infant’s siblings, giving the appearance of integrating the hospitalized infant into the family [34]. In addition, mothers adjusted their emotions and used adaptive strategies to manage the pumping process, in which troubling factors related to expression, including the time element involved in pumping, issues with the breast pump, diversionary tactics during pumping, the pumping environment, the efficiency of milk removal, and physical sensations, affected mothers’ experiences while expressing [37].

##### Avoidance of difficulties

Many teenaged mothers encountered barriers to breast milk provision; however, owing to social and emotional immaturity, teen mothers chose to hide difficulties. When members of the lactation team asked the mothers if they had lactation problems or needed help, the majority of them responded with some variation of the statement “*Everything’s fine*” [39]. In addition, some mothers independently decreased the daily pumping frequency for various reasons, noticing a significant reduction in their supply, yet when asked about their pumped milk volume versus their goals, they answered “*Everything’s fine. I don’t have any questions or concerns*” [39]. Reluctance to seek support from NICU breastfeeding peer counselors (BPC) was attributed to the fear of being judged for not adhering to a breast pump schedule, and feeling embarrassed about exposing their breasts for assistance with breast pump use or breastfeeding, “*You see people just whipping it out. Well, no thanks! No baby of mine is doing that anywhere!*” [39].

## Discussion

This systematic review analyzed and synthesized all qualitative evidence on mothers’ experiences of breastmilk expression during separation from hospitalized infants. It provides insight into maternal experiences related to milk expression within families, healthcare professionals, and policymakers. Additionally, it can support the future development and implementation of specific in-hospital strategies for milk expression. It revealed four synthesized findings related to purpose and motivation, physical and emotional experiences, barriers factors, and coping styles. This section delves into the results for each focal question.

Almost all the participants chose to express milk driven by extrinsic motivation. According to self-determination theory (SDT), motivation is classified as intrinsic or extrinsic, depending on the level of an individual’s autonomy [40]. Intrinsic motivation refers to individuals participating in activities for the activities themselves, without any interest in or expectation of external outcomes [41], while extrinsic motivation is driven by interests other than the activity itself [40]. When providing expressed milk, mothers may attain external outcomes, such as fulfilling emotional needs, shaping self-perception, and establishing self-identity. Despite lacking personal interest or pleasure, mothers still chose to engage in this activity.

However, external motivation cannot create a lasting and profound impact [40]. It’s necessary to take measures to intensify the intrinsic motivation of mothers separated from their hospitalized infants. Evidence has indicated that mothers with intrinsic motivation breastfeed their infants for longer than those with extrinsic motivation [42]. Motivational interviewing (MI) is an instructional, advisee-centered approach that can strengthen a person’s intrinsic motivation and develop their autonomy and competence so that advisees can explore and resolve ambivalence, eventually eliciting behavioral change [43]. When MI is applied to breastfeeding, supporting breastfeeding self-efficacy is a basic principle and a crucial factor in facilitating change [44]. Naroee et al. [45] suggested that MI has a significantly positive impact on enhancing breastfeeding self-efficacy and continuation. In future, SDT and MI can be applied in combination to enhance breastfeeding autonomy and intensify the intrinsic motivation, resulting in longer breastfeeding duration.

Physical exhaustion caused by a strict milk expression schedule was a common topic. In China, guidelines for breastfeeding in neonates in hospital recommend that mothers should express milk every 2–3 h and a minimum of once at night [8]. However, it was difficult for mothers to maintain the motivation to express breast milk, especially during the night. This is important as more milk is produced at night due to the higher night-time level of prolactin; hence expression at night is optimal [46, 47]. A study showed that when fathers were educated about the importance of early, frequent, and effective expression, they would understand the practice and feel motivated to remind mothers to express milk during the middle of the night [48]. Therefore, fathers should be encouraged to provide breastfeeding support and accompany mothers.

Mothers described milk expression as emotionally taxing and a paradoxical experience. They viewed milk expression as the best way to connect with their children, but felt a sense of separation when using a “cold machine”. Pumping milk next to their babies provides a sense of closeness, but in some hospitals in China, mothers have no access to the NICU, making physical proximity impossible. Visualizing one’s infant while expressing is a novel method of supporting lactating mothers. When using videoconferencing with the hospitalized neonates while expressing, mothers perceived better expression of milk and felt connected to the infants [49]. This method could also improve the rate of breast milk provision at NICU discharge [50]. In addition, WeChat, a popular social networking software in China, has been used to send mothers photos of babies who were fed with breast milk, which motivated them to express breast milk early, frequently, and effectively [48]. Webcams are utilized in a limited number of German NICUs, enabling parents to monitor their infants during hospitalization. Mothers reported that the technology increased their sense of closeness and their ability to express breast milk more frequently and in larger volumes. It was also perceived to be more beneficial than receiving support from a photo or recorded video of their infants [51]. But further research is necessary to adapt virtual visits for implementation in diverse cultural contexts.

Additionally, mothers were stressed about the absence of breast milk. This was more evident in those who compared themselves with others who could maintain their milk supply, suggesting that collective-use refrigerators should be avoided and opaque milk containers should be used [32]. Negative emotions might adversely affect the secretion of oxytocin and prolactin, resulting in a reduction of milk supply [52]; in turn, the decreased milk aggravated the level of stress, leading to a vicious circle. A positive maternal emotional experience of feeding was associated with better breastfeeding outcomes [53]. Further research is needed to identify effective interventions to provide emotionally support to mothers of hospitalized infants, rather than focusing primarily on how much milk mothers can produce.

Obstacles to milk expression were identified in this review. Mothers’ decision to express milk was affected by their incorrect perceptions and those of their families about breast milk and formula. This may be associated with the inappropriate marketing of breast milk substitutes [54], and, therefore, full compliance with international and local regulations is needed in advertising, along with the development of a better supervision mechanism [55]. Nurses as the main health educators should offer all-around educational content on breastfeeding, helping mothers understand the reality of breast milk substitute advertising and reinforcing the importance of human milk for hospitalized neonates. However, our results found that nurses paid less attention to mothers who were separated from their infants than to those who were not. As a result, any effort to promote milk expression must consider the knowledge and attitudes of not only mothers and their families but also the healthcare professionals on the postnatal ward.

Mothers also encountered practical barriers that diminished their ability to express milk. Milk expression is the only strategy available to breastfeeding during separation, but it is not as effective in establishing and maintaining an adequate milk supply as suckling an infant at the breast [23]. Neo-BFHI recommends that the facility provides family integrated care (FICare) to enable mothers and infants to remain together 24 h a day [56]. Some countries have implemented FICare in NICUs, wherein families are afforded family rooms equipped with amenities such as a refrigerator, air conditioning, a bed (or sofa), and breastfeeding tools [57]. However, an international questionnaire of neo-BFHI compliance in 917 neonatal units from 36 countries revealed lower implementation of FICare in the NICU [58]. Consequently, there is a need for policies in Chinese NICUs that allow and encourage mothers and hospitalized infants to remain together and promote kangaroo care to establish breastfeeding and improve mother-infant emotional closeness.

This review also demonstrated positive and negative methods used by mothers to cope with milk expression. Consistent with previous study results [59], mothers required significant ongoing informational and emotional support from their partners and from healthcare professionals. Social support has been associated with continued milk expression during the hospital stay of premature infants [60]. However, most mothers perceived support as being insufficient, together with a perception of not being supervised by nurses, and they failed to express milk, although they were shown pumping and hand-expression skills [30]. As the research by Feng et al. showed, mothers struggled to remember things the nurses had said to them only once, meaning that repeating the educational content is necessary [61]. Nurses were too busy to offer one-on-one guidance, increasingly prompting calls for group or mother-directed education [18]. The support of husbands was also key to mothers insisting on milk expression. Mothers described it as highly reassuring, and they appreciated receiving assistance from their partners while expressing milk so that partners could take turns with practical things and help to remember the schedule for milk expression [62]. The process could also be easier if the husband lent a hand when transporting the expressed milk to the hospital [61]. Some mothers dealt with the challenges through self-regulation and adaptation, usually driven by extrinsic motivation, but their efforts need to be recognized and appreciated. We also identified a negative coping method in the teen mothers in that they tended to hide difficulties, outwardly portraying themselves as being in control [39]. Greater attention to teen mothers is suggested, and a matched teen BPC with similar experiences may be more effective in helping teen mothers with barriers to milk expression.

## Conclusions

This systematic review provides insight into mothers’ experiences of breastmilk expression during separation from hospitalized infants. The results indicate that almost all mothers started to express breast milk driven by extrinsic motivation. During the initiation and maintenance of milk expression, they faced many challenges and experienced diverse negative somatic and emotional feelings. The meta-aggregation reflected how mothers had significant needs for practical advice, emotional support, and education from healthcare professionals, as well as needing encouragement and assistance from families, especially their partners. The research revealed that the educational content on milk expression and breastfeeding that is imparted to mothers should be enriched, and should be expanded to the family, community, and breastfeeding knowledge providers working in the postnatal ward. Future research should explore strategies to cope with negative emotional responses, activate social networks, provide respite to mothers, offer practical strategies for managing milk expression, and support positive emotional responses and coping styles.

### Strengths and limitations

This systematic review of 13 qualitative studies is the first to synthesize the qualitative research on experience of breastmilk expression in mothers who are separated from their hospitalized infants. It was rigorously conducted by researchers trained in evidence-based nursing, contributing to a more in-depth and comprehensive understanding of mothers’ experiences of milk expression during separation from hospitalized infants. Qualitative research is more concerned with personal experiences and feelings in a particular situation, synthesizing several relevant qualitative studies using meta-aggregation can offer broad and multiple perspectives as well as evidence of contradictory viewpoints that might otherwise be missed when considering a single study alone, so as to guide clinical decision-making.

There were several limitations in this systematic review. First, the research topic has been widely explored in the USA, although limited in other countries. There is a need to actively explore mothers’ experiences of breast milk expression during separation from their hospitalized infants in regions with relatively high rates of mother-infant separation, such as China. Additionally, although a systematic search was conducted using appropriate search strategies, according to the eligible criteria, only qualitative research or mixed-method studies from which qualitative data could be extracted were included. Only articles written in Chinese or English and published in indexed journals were included, and gray literature and dissertations were not searched. The omittance may have caused information bias. Finally, the included studies were of good quality but, except for one study, they did not report the researchers’ cultural or theoretical background, which may have had an impact on study findings.

### Electronic supplementary material

Below is the link to the electronic supplementary material.

Supplementary Material 1

## Data Availability

Other materials relevant to this review can be found in Supplementary Material. And the raw data supporting the conclusions of this article will be made available by the authors, without undue reservation.

## Abbreviations

NICU: Neonatal Intensive Care Unit
LBW: Low Birth Weight
VLBW: Very Low Birth Weight
Neo-BFHI: Baby Friendly Hospital Initiative for Neonatal Units
SDT: Self-determination Theory
MI: Motivational Interviewing
FICare: Family Integrated Care
