# Supplementary material for: Mothers’ experiences of breast milk expression during separation from their hospitalized infants: a systematic review of qualitative evidence
Source: BMC Pregnancy Childbirth. 2024 Feb 10;24:124. doi: 10.1186/s12884-024-06323-3 (PMC10858471; doi:10.1186/s12884-024-06323-3)
Supplement: Supplementary file 1 — Supplementary Material 1 [file 12884_2024_6323_MOESM1_ESM.docx]

Supplementary Material

**Mothers’ experiences of breast milk expression during separation from their hospitalized infants: A systematic review of** **qualitative evidence**

**Xuemei Li^1^**^†^**, Yongqi Li^2^**^†^**,** **Lin Qian^3^**^†^**, Peng Han^3^, Haoxue Feng^1^, Hui Jiang^4*^**

^†^ Xuemei Li, Yongqi Li and Lin Qian contributed equally to this work and share first authorship.

*** Correspondence:** Hui Jiang: jianghuitest@163.com

**Supplementary Table 1.**

**ENTREQ checklist (Enhancing transparency in reporting the synthesis of qualitative research）**

| **No. Item** | **Guide and description** | **Reported on Page** |
| --- | --- | --- |
| 1. Aim | This systematic review aimed to interpret mothers’ experiences and perceptions while expressing during separation from their hospitalized infants, through critically appraising and synthesizing the qualitative evidence. In particular, the review may deepen the understanding of mothers’ experiences of milk expression among families, health professionals, and policymakers. What’s more, it can aid the future development and implementation of targeted in-hospital strategies for milk expression. | P3 |
| 2. Synthesis methodology | The JBI meta-aggregation approach was used to synthesize the data. Meta-aggregation is grounded in the philosophical traditions of pragmatism and Husserlian transcendental phenomenology, which is the most transparent and widely accepted methodology of all available for qualitative study synthesis for constructing high-quality systematic reviews of qualitative research. This approach accurately and reliably presents the findings and exemplar quotes from original study, categorizing similar phenomena together, and constructing synthesis statements. | P4-5 |
| 3. Approach to searching | The search was pre-planned. Comprehensive search strategies were undertaken to seek all available studies. Sample search strategy for PubMed was presented in Fig. 1. | P3; Fig. 1 |
| 4. Inclusion criteria | Inclusion criteria:  Studies were included according to the following:  i) Participant (P). These were mothers who were separated from their babies that were admitted to a NICU for various reasons such as preterm and low birth weight.  ii) Interest of phenomena (I). Studies were included that focused on mothers’ experiences when they expressed breast milk by hand expression (manual expression), hand-powered pumping (hand pumping or manual pumping), electric-powered pumping (breast pumping), and hands-on pumping (combining hand expression and breast pumping).  iii) Context (Co). Included studies were those performed during mother-infant separation with the infants in the NICU.  iv) Study design (S). Qualitative research and mixed-method studies from which the qualitative part could be extracted were included. Studies were included that used any qualitative methodology, including but not limited to phenomenology, grounded theory, case studies, action research, ethnography, and feminist research.  Exclusion criteria:  i) Studies with qualitative data that were analyzed using quantitative methods.  ii) Duplicate and unavailable full-text literature.  iii) non-English or Chinese literature.  iv) Research not published in peer-reviewed journals, case reports, conference proceedings, poster abstracts, and theses.  v) Systematic reviews and other reviews. We reviewed their references to identify possible relevant studies. | P3 |
| 5. Data sources | We systematically searched 12 electronic databases, including eight English language databases: PubMed, Web of Science Core Collection (via ISI Web of Science), MEDLINE (via ISI Web of Science), Cochrane Library, LWW (via OVID), CINAHL Complete (via EBSCO), Scopus, and ScienceDirect, and four Chinese databases: China National Knowledge Infrastructure (CNKI), Wanfang Database (CECDB), VIP Database, and China Biomedical Database (CBM). Results were limited to journal articles written in English or Chinese and published before 1 December 2022. The references of each qualifying paper were searched manually to identify further relevant studies.  The decision to conduct our research only in these databases was based on an initial scoping search that the most relevant studies for the topic studies can be found in these sources. | P3 |
| 6. Electronic Search strategy | The query included five groups of keywords and MeSH terms combined with Boolean operators: (1) (intensive care units, neonatal*), (infant, premature*), premature birth*, preterm infants, premature infant, (ICU, newborn), (ICU, neonatal), NICU, low birth weight infants, mother-infant separation, maternal separation; (2) mothers*, maternal, maternity; (3) breast milk expression*, human milk provision, expressed breast milk, milk expression, hand expression, manual expression, breast pumping, hands-on pumping; (4) emotions*, perception*, thinking*, attitude*, thoughts, feelings, experience, perspectives, views, point; (5) qualitative research*, hermeneutics*, anthropology, cultural*, feminism*, grounded theory*, focus group*, interviews as topic*, narration*, ethnography, case study, content analysis, qualitative method, phenomenology, descriptive study, exploratory, participant observation, qualitative study, thematic analysis, interview, narrative. A separate search strategy was designed and optimized for different databases. The sample search strategy for PubMed is presented in Figure 1. | P3; Fig. 1 |
| 7. Study screening methods | Study selection was tracked and reported using the PRISMA standards. All records obtained in the initial search were imported into EndNote 20, and duplicate entries were removed. Then two trained reviewers (XL, YL) independently screened the literature according to the inclusion and exclusion criteria. Initially, titles and abstracts were assessed for eligibility. If eligibility was unclear after screening the abstract, the full text was read for further evaluation. The third reviewer (PH) was engaged in discussion if disagreements arose at any stage of the evaluation. | P4; Fig. 2 |
| 8. Study characteristics | Table 2 presents the characteristics of the included studies (the author, publication year, country or region, research aim, research design, method of data collection, sampling and data analysis, participants, and main research results of each qualified study). | P5; Table 2 |
| 9. Study selection results | The database search generated 600 records. Eliminating 72 duplicates left 528 unique records for screening. Then, by reading the titles and abstracts, 509 articles were excluded that were not relevant to the topic or subject, or were not qualitative research. Finally, 13 articles were included in the qualitative synthesis after reading the full text and quality appraisal. The detailed search and screening process is illustrated in Figure 2. | P5; Fig. 2 |
| 10. Rationale for appraisal | A critical appraisal of the methodological quality was performed on the 13 included studies by two trained reviewers (XL, YL) independently, using the JBI Qualitative Assessment and Review Instrument. When the evaluation results conflicted, the third researcher (PH) decided. Ten items were evaluated with “yes” (the study fulfilled the domain criteria), “no” (the study did not fulfill the domain criteria), “unclear” (the study’s adherence to certain domain criteria could not be conclusively proven), or “not applicable”. The reviewers finally included the articles that achieved a minimum of 60% “yes”, a criterion identified by the reviewers to guarantee the study showed acceptable quality. The quality of the study was considered acceptable if 60% of the items answered “yes”, good if 70%–90% of the items answered “yes”, and high if 100% of the items answered “yes”. | P4 |
| 11. Appraisal items | JBI Qualitative Assessment and Review Instrument. | P4; Table 1 |
| 1. Appraisal process | Appraisal was conducted independently by two trained independent reviewers (XL, YL). When the evaluation results conflicted, the third researcher (PH) decided. | P4 |
| 13. Appraisal results | All 13 eligible studies were of good quality. The results of the methodological quality appraisal are presented in Table 1. | P5; Table 1 |
| 14. Data extraction | First, dual data extraction was conducted, including the author, publication year, country or region, research aim, research design, method of data collection, sampling and data analysis, participants, and main research results of each qualified study. Second, study findings were extracted by fully grasping the meaning of each eligible study. A finding was defined as a verbatim extract of the author’s analytical interpretation of the data with illustrations. Two reviewers (XL, YL) independently evaluated the plausibility of each finding and identified them into three levels: (1) Unequivocal (U): the finding was supported by an accompanying illustration that was beyond reasonable doubt and therefore the finding was not open to challenge; (2) Equivocal (E): there was a lack of clear association between the finding and the accompanying illustration, and therefore the finding was open to challenge; (3) Not Supported (NS): the finding was not supported by the illustration. Only unequivocal and equivocal findings were included. | P4-5; Supplementary Table 2 |
| 15. Software | Endnote 20 software was used. | P4 |
| 16. Number of reviewers | 3 reviewers (XL, YL, PH) | Three reviews |
| 17. Coding | JBI meta-aggregation did not use the technique of coding. | NA |
| 18. Study comparison | The extracted findings that possessed similarity in meaning or addressed a similar phenomenon together were summarized to form new categories. Ultimately, these categories were subjected to further synthesis to generate more comprehensive findings, known as synthesized findings. | P5 |
| 19. Derivation of themes | The process of deriving the themes or constructs was inductive. Only unequivocal and equivocal findings were included. Findings that possessed similarity in meaning or addressed a similar phenomenon together were summarize to form new categories. Ultimately, these categories were subjected to further synthesis to generate more comprehensive findings, known as synthesized findings. | P5; Supplementary Table 3 |
| 20. Quotations | Supplementary Table 2 provided findings and quotations from the primary studies to illustrate themes and constructs, and identify whether the quotations were participant quotations of the author’s interpretation. | Supplementary Table 2 |
| 21.Synthesis output | Synthesis output is presented in Supplementary Table 3.  A total of 61 primary findings with illustrations, classified as equivocal or unequivocal, were extracted from the 13 included studies. The findings were generalized into 16 categories based on their meaning. From the 16 categories, four synthesized findings emerged: purpose and motivation, physical and emotional experiences, barrier factors, and coping styles. | P5; Supplementary Table 3 |

**Supplementary Table 2.**

**Findings extracted from the included studies with illustration**

| **Brodsgaard et al. (2022)** | |
| --- | --- |
| Finding 1 | Expressing human milk is essential for breastfeeding (Equivocal) |
| Illustration | “I didn’t get information about starting milk expression at the delivery unit, so I started late, then I got sick with constipation and infection, and then I didn’t express milk as often as I should. ” (Participant 7, P563) |
|  | Expressing milk and lactation to compensate for the infant's prematurity (Unequivocal) |
| **Fernández Medina et al. (2019)** | |
| Finding 2 | The extremely preterm birth and the decision to provide mother’s own milk (Unequivocal) |
| Illustration | “When circumstances have robbed you of a natural birth and a full-term infant, you don’t have much more to offer.” (Participant 11, P351)  “When the pediatrician came to my room I was bewildered and thought: Do I have to express milk now? I was not strong enough to try it, but when he told me that my milk was the best medicine for my infant, I automatically said yes.” (Participant 15, P353) |
| Finding 3 | Feelings of bereavement (Unequivocal) |
| Illustration | “In less than 10 minutes my infant was no longer inside me, and I had not heard anything … and there I was, alone.... Why would I express milk if I had not even seen my baby was alive? I had pre-eclampsia and I did not feel well. I could not visit my infant until 30 hours later .... ” (Participant 5, P353) |
| Finding 4 | Lack of support and supervision (Unequivocal) |
| Illustration | “The nurse gave me the information and offered to bring me a breast pump to the room. The first time I used it I felt a horrible pain … and not a drop came out of my breast. I felt so disappointed that I did not want to try again.” (Participant 3, P353)  “ Searching for information online, I found that I had not placed the cup to my breast properly.” (Participant 14, P353) |
| Finding 5 | Mother–infant separation (Unequivocal) |
| Illustration | “The first days at home after hospital discharge, due to my physical condition and distance hospital from family home, I could not do anything, just express milk for my husband to take it to the NICU ... even though I was with the breast pump all day, I could not express anything ... I could not see my infant and I could not give him my milk either.” (Participant 12, P354) |
| Finding 6 | Complications (Unequivocal) |
|  | “The problem came when my infant began to have serious health problems ... I missed the timetable to express milk ... I did not want to touch him or have skin-to-skin contact, and this caused the milk supply to go down.... ”(Participant 3, P354) |
| Finding 7 | My job was to make milk (Unequivocal) |
|  | “I felt a bit overwhelmed because I felt bad and also I did not have enough milk ... and I had to force myself because the nurses said I had to have more milk.... There was great pressure to know that my milk was of vital importance to my infant.... ”(Participant 1, P354-355) |
| **Yang et al. (2019)** | |
| Finding 8 | Providing breast milk is a way to identify as a mother (Unequivocal) |
| Illustration | “It’s the breasts starting to go up and milk oozing out that let me think I am a mother.” (P3)  “Preterm infants are more fragile than full-term infants. The only thing mothers can do is to express more milk for them.” (P3) |
| Finding 9 | Perceptions and intentions (Unequivocal) |
| Illustration | “… didn’t want to breastfeed.” (P3)  “I don’t think breast milk can provide enough nutrition for preterm infants. They need large amounts of nutrients to get the weight gain. But the breast milk looks pretty dilute and the color is clear. It seems that breast milk has fewer nutrients than the formula.” (P3-P4)  “Both the amount and quality of breast milk are not good enough. The child will grow slowly if she only [gets] breast milk.” (P4)  “I think that formula is more nutritious for the [baby’s] weight gain …breast milk is definitely not as good as formula.” (P4)  “I am afraid that the quality of my milk is not good, and the amount is inadequate… due to my wrong diet, which will affect the health of my kid.” (P4)  “… a lot of my friends and colleagues don’t breastfeed.” (P4)  “My siblings fed their children with formula.” (P4)  “Formula is also very good for the child’s development.” (P4)  “My parents asked me not to breastfeed my baby because formula feeding is easier than breastfeeding.” (P4)  “When my first kid was born, I [wanted] to breastfeed. However, I didn’t have enough milk… [and breastfeeding] … caused me fatty [weight gain]. [So] I’m not insisting on it now.” (P4)  “…formula feeding is much easier for the mother; I don’t want to be so tired.” (P4)  “… as long as the child is discharged from the hospital, whatever he eats does not matter.” (P4)  “… because of my work, I don’t want to breastfeed.” (P4)  “… it is not easy to ensure adequate milk for the kids without getting fatty [gaining weight].” (P4)  “… may cause… sagging of the breasts.” (P4) |
| Finding 10 | Milk expression makes mothers exhausted (Unequivocal) |
| Illustration | “…a nervous breakdown.” (P4)  “… too tired at night. No matter how late at night, as long as the alarm clock sounds, I have to sit up to express milk… [it] needs perseverance.” (P4) |
| Finding 11 | Health professional support on breastfeeding are urgently needed (Unequivocal) |
| Illustration | “ Of course, I believe the healthcare providers. They are the experts.” (P4)  “I trust doctors and nurses.” (P4)  “… doctors and nurses are too busy…[and] one-on-one guidance is not feasible.” (P4)  “videos made by the hospitals… [would be] more useful and reliable than the lay person [as health professionals] can…correct the wrong ideas of the elderly members of my family and avoid the quarrels between us about feeding my baby.” (P4)  “professional books published by doctors and nurses would be very helpful.” (P4)  “official booklet is more convincing than... hearsay.” (P4)  “… in the maternity ward, the nurse asked me to watch a video about newborn care. It was very helpful.” (P4)  “Milk expression techniques are all learned online.” (P4)  “I have been exploring [the internet] by myself.” (P4)  “There are many people in the WeChat group, but no one answered my questions, unlike one-on-one guidance where you will definitely get an answer.” (P5)  “I haven’t found a book …on breastfeeding for preterm infants.” (P5)  “… too busy to answer questions [and were] ashamed to increase the burden on them.” (P5)  “My husband and I once argued. We finally found that we got the ideas from different books. We [didn’t] know which statement is right. It [was] confusing.” (P5)  “Other mothers’ advice is not completely suitable for my kid.” (P5) |
| **Bujold et al. (2018)** | |
| Finding 12 | Closeness and Positive Feelings (Unequivocal) |
| Illustration | “When I’m with my baby, ... it’s very motivating to have your baby close, and sometimes I even have him on me while expressing my milk...” (Participant 5, P41)  “I still feel close to my baby because I went to see him this morning… When I think of him… pleasant thoughts come to me. I know he’s okay… that he’ll have good milk.” (Participant 6, P41) |
| Finding 13 | Considering milk expression as a way to feel useful as a mother (Unequivocal) |
| Illustration | “I think it’s so important to give him his milk …, this is the only way you can feel like you are close to the child because he is sick and he is away in the NICU and pumping and giving him milk is like a way to feel like you are important for your child. You feel you’re a mother.” (Participant 9, P41) |
| Finding 14 | Using self-talk (Unequivocal) |
| Illustration | “It’s motivating to remind myself that I’m doing this for her , when I see her and I know she’s going to drink this milk.” (Participant 8, P42) |
| Finding 15 | Thinking about their infant (Unequivocal) |
| Illustration | “I like to think about my baby, it brings positive thoughts. I know … he will get good milk.” (Participant 6, P42) |
| Finding 16 | Reframing Milk Expression as a Part of the Lactation Process (Unequivocal) |
| Illustration | “Pumping my milk, well it is part of the breastfeeding process.” (Participant 1, P42) |
| Finding 17 | Integrating Milk Expression Into Daily Life (Equivocal) |
| Illustration | “I sit at home and I can help my other children while telling myself that I’m helping the one who isn’t present at that moment.” (Participant 6, P42) |
| Finding 18 | Using Distractions to Pass the Time (Unequivocal) |
| Illustration | “I’m alone in a small room so I check my phone, check social media, or play games… findings ways to pass time because I need to pump for 15 minutes. But I will go back to my baby right after.” (Participant 1, P42) |
| Finding 19 | Seeking and Using Support (Unequivocal) |
| Illustration | “He [husband] is encouraging me a lot. He supports and understands and appreciates what I’m doing for our child. This is for me the power to continue pumping.” (Participant 9, P43) |
| Finding 20 | Having a Negative Perception of Milk Production (Unequivocal) |
| Illustration | “I’m not feeling very close, just feeling stressed out that I’m not producing enough milk.” (Participant 13, P43) |
| Finding 21 | Considering That Milk Expression Is Not Breastfeeding (Unequivocal) |
| Illustration | “Of course, I’ve never experienced it, so it’s hard to say, but it’s less appealing to express your milk at night than to wake up to breastfeed your baby.” (Participant 1, P43)  “These days, I’m not feeling emotionally connected. In the beginning, I remember really feeling like I had a mission and I was doing this [pumping] for my infant and it was one of the only things that I could really … do for him …” (Participant 11, P43) |
| Finding 22 | An Exhausting Schedule (Unequivocal) |
| Illustration | “That’s really when I feel the most alone, the furthest away. I don’t think there’s really anything to help at that hour. You need will power to get up, express your milk. Then you tell yourself, I’m tired, why am I doing this?” (Participant 6, P43) |
| Finding 23 | Lacking Adequate Support (Unequivocal) |
| Illustration | “I’m at the hospital every day with my daughter… I understand that they’re there for the baby’s needs, but we are here all day too. We have work to do so that this baby can grow, and I think we aren’t helped enough.” (Participant 7, 44). |
| Finding 24 | Location and Environment (Unequivocal) |
| Illustration | “I’m at home pumping… I feel isolated from our infant because we’re not there. He’s over at the hospital and we’re here and it feels strange to be doing something for him when he’s not in our home.” (Participant 13, P44)  “I pump my milk next to my baby’s isolette. I’m able to look at my baby. It’s a nice moment for me, I feel very close to him.” (Participant 1, P44) |
| **Parker et al. (2018)** | |
| Finding 25 | Breast milk benefits for premature infants (Unequivocal) |
| Illustration | “I know it’s very important for all children to have breastmilk; especially for children who are born so small, with few defenses.” (P5) |
| Finding 26 | Emotional connection (Unequivocal) |
| Illustration | “I was happy because I’ve always liked to breastfeed my kids, to me that’s a very beautiful experience.” (P5) |
| Finding 27 | Pumping is not the same as breastfeeding (Unequivocal) |
| Illustration | “ That [the pump] is not a nice thing, nobody likes to get up and pump.” (P5)  “I think breastfeeding and pumping are two different things...like if I would have breastfed her that probably would have been a lot easier than just pumping.” (P5) |
| Finding 28 | Repetition, exhaustion (Unequivocal) |
| Illustration | “I have to pump every two hours and get it done, but there is always something to do, getting the house together for the baby, moving furniture, vacuuming, sanitizing, dusting, folding and rearranging and do it all over again.” (P5) |
| Finding 29 | Hospital providers are an important source of support, when sufficient time is spent to address ongoing issues (Unequivocal) |
| Illustration | “But it was very tough and I felt really bad. I didn’t know how to help her. But the doctors told me everything was going to be fine. They comforted me and told me they had all the necessary equipment and specialists to help her survive.” (P5)  “The nurses—they’re like family. Because they know your name, they know your needs. They’re great support. I think the staff is awesome.” (P5)  “I didn’t really have anybody to talk to. The lactation teacher came and spoke to me, but it was like, a five second conversation. They didn’t say much to me about what to do.” (P5) |
| Finding 30 | Logistical and financial challenges (Unequivocal) |
| Illustration | “I spent so much of my money on cabs, coming back and forth to the hospital , and I had to space out times. I didn’t have the money to go back and forth. I think that’s the worst part of them being in the hospital, is transportation. That’s it.” (P6)  “[From a Spanish-speaking mother] I had to travel by train, because I didn’t know my way around here. So I had to learn. I had to learn to ride the train. I had to go to the hospital every day and always someone different at the entrance, and having to tell them in English that I was there to see my baby, that’s something quite difficult.” (P6) |
| Finding 31 | Stress of mother–infant Separation (Unequivocal) |
| Illustration | “I could not skip seeing her one day, so that caused a lot of stress. Not only financially, but also physically, in my health. It was exhausting to go back and forth every day.” (P6)  “So it wasn’t easy for me. I did get a little depressed. Sometimes I would leave the place [NICU] crying. Many people, or the nurses would say. everything is fine, but as a mom, you don’t want to leave your children there. It’s hard to see them there. You want to have them with you, you want to have them at home, give them love and care, not leave them somewhere else. So, it was like dividing myself. Sometimes I was with her, sometimes I was with my other daughter. And that time was very hard for me.” (P6)  “Sometimes I think it [lack of milk production] was because of some stress, because. it was quite a complicated experience. I had just arrived here [the US]. I had him in the hospital. it was quite hard. So I think I run out of milk because of the stress.” (P6) |
| **Bower et al. (2017)** | |
| Finding 32 | I was heartbroken (Unequivocal) |
| Illustration | “I would come in with maybe a couple of drops in each bottle and be really embarrassed and depressed about it.” (P191)  “There was that decision to make . . . do I want to be with my son or do I want to feel terrible and make a little bit more milk . . . and then the emotional stress of that would affect how much I made.” (P191)  “[Pumping] . . . is hard to do, and then the stress of having a sick child and putting that added stress on yourself. . . . Basically, you know that it’s something you have to do but you’re not necessarily in a good place, mentally, emotionally, physically.” (P191)  “I would do everything she told me to do and—nothing . . . and . . . I was like, my back was against the wall, ‘cause all she could tell me was, you know, ‘Make sure you’re eating right, getting rest, pumping every 3 hours, faithfully, plenty of water, no stressing.’ . . . Besides the stressing part, ‘cause I had no control over it, I was doing everything, and nothing.” (P191)  “I was trying to breastfeed until he was released from the NICU. That was the goal. At least until he was released. But I couldn’t—it was painful. It wasn’t coming out how it was supposed to. . . . I was stressing my body out, stressing myself out, and I was like, that’s it. I can’t. It’s like, I can’t. There’s nothing producing. That’s when I decided to stop.” (P191) |
| Finding 33 | Pumping is a full-time thing (Unequivocal) |
| Illustration | “With family, I have to take my time away from them to go somewhere to be able to pump or if I don’t take my pump with me that just makes me lose more milk. The longer you go without pumping, the less milk you produce, so it just takes a lot of time away from people.” (P191)  “Just having to stop what you’re doing . . . then my son always, like, wants to play with me and stuff when I’m pumping and I have to tell him no, so that can be very stressful.” (P191)  “Pumping is the pits. I’ll be honest—it is not fun. Three hours . . . it seems like a long time but it’s not, so there’s always the stress of, oh my god, it’s almost time . . . I’ve gotta pump.” (P191)  “It’s just uncomfortable and, I mean, it interferes with your schedule ‘cause, like, every 3 hours, you have to stop whatever you’re doing or hurry home . . . so you can pump.” (P192)  “Sometimes, like, I have to skip something for pumping and I have to skip pumping like that and that is minor challenges but having baby here is stressful.” (P192) |
| Finding 34 | I literally sacrificed nights (Unequivocal) |
| Illustration | “When I first started again, I was tired but I wasn’t as tired, so I would literally get up, at first, every 3 hours and pump. Which, when you do that, you think, oh well, that’s great, I’ll get 3 hours of sleep and I’ll get up to pump and I’ll get 3 hours of sleep. Well it doesn’t work that way.” (P192)  “Sometimes I skip ‘cause I just oversleep and be so tired. I think that’s why my milk supply has gotten a little lower, but I’m trying to not do that anymore.” (P192)  “Thirty minutes, every 2 to 3 hours a day. . . . If he was home, it would be a lot easier . . . it doesn’t always take as long to breastfeed him. . . . So with him not being home, it, sometimes I often forget, like, exactly to pump on time because he’s not there to feed.” (P192)  “It does get harder, though, because he’s not with me, so you kind of have to have a scheduled time, sit down, and pump milk and still go on do your day. It takes a lot of dedication.” (P192) |
| **Froh et al. (2017)** | |
| Finding 35 | Hopeful for breastfeeding (Unequivocal) |
| Illustration | “And maybe that will happen, and if it does that’s great. I mean, I’d love to see . . . hopefully I’ll have the opportunity to try when he’s ready to.” (Hannah, P5)  “But hopeful . . . to hopefully get to that point, continuing to pump to keep my supply up for when she is ready to breastfeed. That’s a huge motivator in itself.” (Sandra, P5) |
| Finding 36 | Latching on . . . to the pump (Unequivocal) |
| Illustration | “The most challenging thing is dealing with it while your child is not here because most people who do breastfeeding have their baby latching on. . . . But you’re latching on a pump as if you’re feeding your baby on a consistent basis, even waking up in the middle of the night acting like you’re feeding this baby.” (Effie, P5)  “My second day when I didn’t get any milk, I looked at pictures of my son as I pumped. I imagined him being there. I imagined myself holding him, and even though that entire day I didn’t get any milk, I pumped consistently every 2 hours, and since then I’ve had a beautiful flow of milk.” (Kenya age 28, P5)  “And she [the lactation consultant] also told me how important it[keep expressing milk and maintain her milk supply] was, which was interesting because I didn’t know it was that key. I mean I would have done it even if she didn’t tell me that because I would have wanted to. In my mind I’m doing it for now the reasons that she told me because it’s so important for these babies to have this. It’s like medicine for them. I’m really doing it[expressing milk] . . . but also I have this motive that I want to be able to have milk when he’s ready to breastfeed.” (Amy , P5)  “I think they’re both the same, right? Because he’s getting potentially . . . the exact same thing; he’s getting the breast milk still. I mean, for me going home it will be much easier obviously if he’s breastfeeding instead of feeding and then me pumping. Obviously that will be the end goal, but for now I’ve already grown accustomed to this lifestyle of pumping for 3 hours and keeping that going, and by no means will he get anything else unless he has to ‘cause of weight gain, but breast milk is what I want him to get—all the time, until he’s at least 1.” (Tessa, P5) |
| Finding 37 | We’ve Already Worked So Hard (Equivocal) |
| Illustration | “He just had surgery 15 days ago, so for him I think as much of this milk that he can get, the better off he’s gonna be in terms of getting sick and being able to get healthy . . .” (Amy, P5)  “I’m also realistic and it’s not about me—it’s about what’s best for him so I’m willing to do whatever. It would be my first choice, but if it doesn’t work, I’ll still pump and give it to him in a bottle.” (Hannah, P5) |
| **Rossman et al. (2017)** | |
| Finding 38 | Wanting to do the right thing (Unequivocal) |
| Illustration | “That’s what a mother’s supposed to do, put her kids first.” (P3)  “I gotta put my own needs aside and just do it[providing human milk] to help him, to do the right thing.” (P3) |
| Finding 39 | Everything’s fine (Unequivocal) |
| Illustration | (decrease the daily frequency of breast pump use for various reasons: )‘‘There are other things at home needing doing’’, ‘‘I just didn’t like that I was wetting up all my shirts’’, ‘‘He don’t like it (HM) no more’’. (P4)  “I just won’t pump for eight hours. It’ll be fine.” (P4)  “Didn’t think about that[how to find time and space to remove and store milk] yet.” (P4)  “Everything’s fine. I don’t have any questions or concerns.” (P4) |
| Finding 40 | Comfort with family versus NICU staff for continued breastfeeding support (Unequivocal) |
| Illustration | (they [teen mothers’mothers])‘‘didn’t like it’’ or ‘‘wouldn’t latch on’’. (P4)  (comments made by teen mothers’ mothers:) ‘‘Why would you want to do that (provide HM)?’’ ‘‘I bet you’re not gonna do it (provide HM).’’ and ‘‘Don’t it hurt?’’ (P4)  “ You see people just whipping it out. Well, no thanks! No baby of mine is doing that anywhere!” |
| **Ikonen et al. (2016)** | |
| Finding 41 | Managing the situation (Equivocal) |
| Illustration | “At the beginning, there were only a few drops[colostrum], but I was told that they are extremely valuable, like medicine for my baby, so I should continue.” (P112)  “in the beginning, a lifeline[breast milk] for the baby.” (P112) |
| Finding 42 | Looking forward to easier breastfeeding (Unequivocal) |
| Illustration | “I’m stubborn and I’ve decided to breastfeed, even if this is the only thing[milk expression] I’m able to cope with.” (P113)  “Beginning by expression was worth it. I was able to breastfeed when the time came for me to do it.” (P113) |
| Finding 43 | Managing daily life (Unequivocal) |
| Illustration | “Daily life revolved around pumping, and I had to schedule all my plans according to it. It was also taxing on my sleep routine.” (P113)  “Maintaining equipment, boiling bottles, sterilization—these took hours and I was constantly extremely tired.” (P113)  “I had kind of a huge and outdated pumping machine. It would have been easier if I had something small and handy that I could easily move, for example, next to my bed.” (P113)  “In the NICUs, there was no peaceful location, nowhere that a mom can feel safe and secure. In [university hospital], the pumping machine was in the parents’ recreation room, and if someone was already occupying the room, you had to a) ask that person to move to another area, b) watch the room until it becomes vacant, or c) leave and endure having swollen breasts.” (P113) |
| Finding 44 | Managing feelings (Unequivocal) |
| Illustration | “I was already used to expressing when my firstborn was a baby, so the second time around, it became ‘routine’ for me.” (P113)  “I didn’t see it [expressing] as weird. Instead, it was the most suitable method for us.” (P114)  “It [expressing] went right. I was proud of my milk supply. I knew it was important for my baby.” (P114)  “Expressing was weird, especially when it was done with the hospital’s strange and huge machine.” (P114)  “The irregularity of milk supply was troubling, and I was immediately afraid that the milk would dry up when it decreased from time to time.” (P114)  “Expressing was frustrating because the milk did not even come out properly. I was severely stressed out and depressed. […] I would have stopped expressing earlier if I knew how hard it was and how the milk kept on decreasing.” (P114) |
| **Rossman et al. (2013)** | |
| Finding 45 | The healing power of milk (Unequivocal) |
| Illustration | “They told me, ‘We cannot clone your milk. No doctor, no nurse, nobody can give him this. Only his mom can give him this.’ So I went on ahead and did it because I knew it was gonna make him better and help him.” (P362)  “it’s[mothers’ milk] life for my baby.” (P362)  “what[mothers’ milk] he needs to grow and be big and strong.” (P362)  “I’m giving him life, medicine, food, and a part of me, all in a feeding every 2 hours.” (P362) |
| Finding 46 | Mitigating complications (Unequivocal) |
| Illustration | “This is life for my baby. Maybe it [providing milk] will help, however it’s gonna happen, with the cyst going away. I’m trying to give him an extra chance at life because, obviously, he needs that.” (P362)  “I want to produce enough so he doesn’t have to have formula. I try to think that this is gonna help prevent future problems. This is his life now and it’s just, okay, let’s see what we can do to keep him healthy and stable.” (P362) |
| Finding 47 | The paradox of providing milk and pumping (Equivocal) |
| Illustration | “The fact that I’m pumping for her—I don’t care for it. If I had a choice I would stop, but the fact that I know that it’s good for her, I do it.” (P363) |
| Finding 48 | The maternal healing proces (Unequivocal) |
| Illustration | “I couldn’t hold her in my uterus long enough.”  “I’m doing for her outside what I couldn’t do for her in my womb.” (P363) |
| Finding 49 | Bonding/connecting through the ritual of providing milk (Unequivocal) |
| Illustration | “I think the breast milk—it’s me. I feel connected ’cause my breast milk is a part of me. I mean, I’m makin’ this milk.” (P363)  “Even if I can’t be there for him every single day, I’m pumping still. So you feel like you’re doing something for him. I just don’t want anything else to get in the way of pumping.” (P363) |
| Finding 50 | Rewards and motivation (Unequivocal) |
| Illustration | “it’s [providing milk] doing something for him, it’s very rewarding and it’s sustaining his life. And he’s gaining weight, so it specially makes you feel good.” (P363)  “Now he’s doing the formula but I’m still pumping. He better now, but I’m still trying. So that what breast milk I bring will help him grow and get better to come home to me.” (P364) |
| **Hurst et al. (2013)** | |
| Finding 51 | A paradoxical experience (Unequivocal) |
| Illustration | “how you’re trying to love your child even though you are attached to a machine . . . it is pretty difficult to describe.” (P369)  “I do look at the pump but I know it’s not my baby, but I think this is for my baby.” (Beth, P369) |
| Finding 52 | Negotiating (Unequivocal) |
| Illustration | “thinking about when the babies come home and I can feed them.” (P370)  “ Oh good, I can’t wait to see what happens next [in the book].” ( they could overcome the “dread” of pumping) (P370)  “I close my eyes and think of money falling from the sky.” (P370) |
| **Sisk et al. (2010)** | |
| Finding 53 | Physical challenges (Unequivocal) |
| Illustration | “Have you ever been on mag[nesium]? Oh my God, you’re loopy . . . I just didn’t care . . . I was just drunk.” (P371)  One mother(delivered by cesarean section) said, “They [the lactation consultants] say to do it every 8 hours,” and another(delivered by cesarean section) said she was told to “pump every 5 hours.” (P371) |
| Finding 54 | Distance and separation (Unequivocal) |
| Illustration | “It was hard to stay on a pumping schedule traveling back and forth to the NICU.” (P371)  “I had to ride the bus from XXX to XXX so that would put me off [my pumping] schedule a bit, because you know the bus doesn’t take you right to the hospital. It has all these other stops . . . but I would try to make it up. Each time when I got [to the hospital] I’d pump a little longer and then I’d try to pump right before I left the hospital.” (P371) |
| Finding 55 | Balancing work and pumping (Unequivocal) |
| Illustration | “I couldn’t just stop what I was doing, . . . if [the factory order] was due that day.” (P372)  “ I was just so disconcerted being in a work environment.” (P372) |
| Finding 56 | Time management (Unequivocal) |
| Illustration | “I really want to take a nap but I need to pump. I really need to eat but I need to pump.” (P372)  “I need to pump but I don’t want to go because I know that they’re [the medical team] going to be here soon and then they wouldn’t be there for 2 hours and . . . your whole day would be thrown off.” (P372) |
| Finding 57 | Attitudes (Unequivocal) |
| Illustration | “not how breastfeeding would feel.” (P372-373)  “I hated waking up during the night just to pump but . . . it’s getting you ready for the baby once he comes home.” (P373)  “They wanted me to pump every 2½ to 3 hours. I didn’t have enough time to do all that.” (P373) |
| **Sweet. (2008)** | |
| Finding 58 | Breastfeeding is initially not a priority (Unequivocal) |
| Illustration | “My main concern was him, you know, even though they say express or whatever, I was never in my room, I was always downstairs, ... and all I’m concerned about is him medically, how he is, you know, not my milk supply, you know.” (Alison, P4)  “... because I didn’t have Joel with me, you don’t have the nurses and all the people kind of giving you all this information, they’re dealing, you know, with the breastfeeding issue [s] with the women whose babies are right next to them. Me without having my baby, I didn’t have, like, the nurses coming in and having chats to me, you know, they just showed me the machine, what I had to do and that was the end of it, you know. ... But, yeah, the whole breastfeeding thing wasn’t a priority or a major issue because my baby wasn’t with me.” (Alison, P5)  “... And I know why they do it, because they want you to focus on that more, because that’s the next step, the milk supply. Like you’ve got over the section ... and now I have to really focus in on the milk.” (Helen, P5) |
| Finding 59 | Breastfeeding as a marker of “good” motherhood (Unequivocal) |
| Illustration | “I probably would have felt quite disappointed, I guess, if I wasn’t able to do it and maybe I would have felt as though I was letting them down a bit or maybe I was inadequate or something like that.” (Sue, P6)  “Yeah, inadequate a bit. Like you weren’t doing what you were supposed to be doing, or getting, that you were a bit of a failure.” (Chris, P6)  “... It is my responsibility, because I want the best for him you know. And my milk is the best for him, there’s no two ways about that, you know, and like I said ... I think every time I have little milk I feel guilty.”(Alison, P6)  “[to be] A better mum than anybody else. Certainly not a bad mum. I tried and I did what I could[lasting longer at breastfeeding than other mothers did] and, you know.” (Julie, P7) |
| Finding 60 | “So much is taken out of your hands” (Unequivocal) |
| Illustration | “And it is good because I think you get that feeling that you’re doing something for your babies, even though they’re not with you. It’s, you know, you just think babies and think milk is for babies and this is my milk and this is what you’re contributing to your babies.” (Julie, P7)  “With a preterm baby, I mean, it’s just, there’s so little you can do for them, you know, so much is taken out of your hands and it’s just something [expressing breast milk], you know, even when I’m not with her, it’s still something I know that I’m doing for her and I guess a lot of the time when I’m expressing at home I’m thinking about her, so, yeah.” (Lisa, P7)  “Plus it’s the only thing you can actually do for them because they are prem, apart from going and sitting and sort of touching them and you know changing their nappy occasionally and having them out for a hold I mean there’s not really much you can do to care for them. But at least if you are giving ... expressing your breast milk, it is something that you know you feel you’re contributing to their well being, I guess.” (Sue, P8)  “[sigh] I feel closer to him than, yeah, I feel closer that he’s having some of my milk, I’m giving him something to help him grow.” (Nicole, P8) |
| Finding 61 | “You have to do it” – Expressing is not a choice (Unequivocal) |
| Illustration | “It’s like in two parts. There’s the expressing part and all that and now there’s this part [at-breast feeding], which is like the normal part. So it’s got, like, a really nasty bit which is the expressing all the time, ... Yeah. It’s horrible. It’s alright, it’s good, but i’s, like, well, you’re going to have to do it, but ... but it’s really hard.” (Helen, P8) |

**Supplementary Table 3.**

**Summary of study finding, categories, and synthesized categories to generate synthesized findings on mothers’experiences when expressing breast milk during separation from their hospitalized infants.**

| **Synthesized finding 1:** **Purpose and motivation** | | | |
| --- | --- | --- | --- |
| Findings  (Credibility Rating) | | Categories | Synthesized  Category |
| 48 | The maternal healing process (Unequivocal) | To alleviate the feeling of guilt | Purpose and motivation |
| 2 | The extremely preterm birth and the decision to provide mother’s own milk (Unequivocal) | For sake of infants’ health |  |
| 25 | Breast milk benefits for premature infants (Unequivocal) |  |  |
| 37 | We’ve Already Worked So Hard(Equivocal) |  |  |
| 38 | Wanting to do the right thing (Unequivocal) |  |  |
| 41 | Managing the situation (Equivocal) |  |  |
| 45 | The healing power of milk (Unequivocal) |  |  |
| 46 | Mitigating complications (Unequivocal) |  |  |
| 50 | Rewards and motivation (Unequivocal) |  |  |
| 26 | Emotional connection (Unequivocal) | To create a bond with infants |  |
| 49 | Bonding/connecting through the ritual of providing milk (Unequivocal) |  |  |
| 8 | Providing breast milk is a way to identify as a mother (Unequivocal) | As a symbol of maternal role |  |
| 13 | Considering milk expression as a way to feel useful as a mother (Unequivocal) |  |  |
| 59 | Breastfeeding as a marker of “good” motherhood (Unequivocal) |  |  |
| 60 | “So much is taken out of your hands” (Unequivocal) |  |  |
| 1 | Expressing human milk is essential for breastfeeding (Equivocal) | For the transition to breastfeeding |  |
| 16 | Reframing Milk Expression as a Part of the Lactation Process (Unequivocal) |  |  |
| 35 | Hopeful for breastfeeding (Unequivocal) |  |  |
| 36 | Latching on ... to the pump(Unequivocal) |  |  |
| 42 | Looking forward to easier breastfeeding (Unequivocal) |  |  |
| 61 | “You have to do it” – Expressing is not a choice(Unequivocal) |  |  |
| **Synthesized finding 2: Physical and emotional experiences** | | | |
| Findings  (Credibility Rating) | | Categories | Synthesized  Category |
| 10 | Milk expression makes mothers exhausted (Unequivocal) | Physical exhaustion | Physical and emotional experiences |
| 22 | An Exhausting Schedule (Unequivocal) |  |  |
| 28 | Repetition, exhaustion (Unequivocal) |  |  |
| 34 | I literally sacrificed nights (Unequivocal) |  |  |
| 3 | Feelings of bereavement (Unequivocal) | Feeling of bereavement |  |
| 7 | My job was to make milk (Unequivocal) | Overwhelmed by stress |  |
| 20 | Having a Negative Perception of Milk Production (Unequivocal) |  |  |
| 31 | Stress of mother–infant Separation (Unequivocal) |  |  |
| 32 | I was heartbroken (Unequivocal) |  |  |
| 33 | Pumping is a full-time thing(Unequivocal) |  |  |
| 12 | Closeness and Positive Feelings (Unequivocal) | Closeness and separation |  |
| 21 | Considering That Milk Expression Is Not Breastfeeding (Unequivocal) |  |  |
| 27 | Pumping is not the same as breastfeeding (Unequivocal) |  |  |
| 24 | Location and Environment (Unequivocal) |  |  |
| 47 | The paradox of providing milk and pumping (Equivocal) | A paradoxical experience |  |
| 51 | A paradoxical experience (Unequivocal) |  |  |
| **Synthesized finding 3: Barrier factors** | | | |
| Findings  (Credibility Rating) | | Categories | Synthesized  Category |
| 6 | Complications (Unequivocal) | Physical condition | Barrier factors |
| 53 | Physical challenges (Unequivocal) |  |  |
| 9 | Perceptions and intentions (Unequivocal) | Perceptions and attitudes |  |
| 40 | Comfort with family versus NICU staff for continued breastfeeding support (Unequivocal) |  |  |
| 44 | Managing feelings (Unequivocal) |  |  |
| 57 | Attitudes (Unequivocal) |  |  |
| 58 | Breastfeeding is initially not a priority (Unequivocal) |  |  |
| 5 | Mother–infant separation (Unequivocal) | Practical obstacles |  |
| 30 | Logistical and financial challenges (Unequivocal) |  |  |
| 43 | Managing daily life (Unequivocal) |  |  |
| 54 | Distance and separation (Unequivocal) |  |  |
| 55 | Balancing work and pumping (Unequivocal) |  |  |
| 56 | Time management (Unequivocal) |  |  |
| **Synthesized finding 4:** **Coping styles** | | | |
| Findings  (Credibility Rating) | | Categories | Synthesized  Category |
| 4 | Lack of support and supervision (Unequivocal) | Seeking social support | Coping styles |
| 11 | Health professional support on breastfeeding are urgently needed (Unequivocal) |  |  |
| 19 | Seeking and Using Support (Unequivocal) |  |  |
| 23 | Lacking Adequate Support (Unequivocal) |  |  |
| 29 | Hospital providers are an important source of support, when sufficient time is spent to address ongoing issues (Unequivocal) |  |  |
| 14 | Using self-talk (Unequivocal) | Self-regulation and adaptation |  |
| 15 | Thinking about their infant (Unequivocal) |  |  |
| 17 | Integrating Milk Expression Into Daily Life (Equivocal) |  |  |
| 18 | Using Distractions to Pass the Time (Unequivocal) |  |  |
| 52 | Negotiating (Unequivocal) |  |  |
| 39 | Everything’s fine (Unequivocal) | Avoidance of difficulties |  |
